# Elevated postoperative carcinoembryonic antigen guides adjuvant chemotherapy for stage II colon cancer: a multicentre cohort retrospective study

**DOI:** 10.1038/s41598-024-55967-w

**Published:** 2024-03-22

**Authors:** Hongjiang Pu, Wei Yang, Mengmei Liu, Xiaolin Pang, Yaxue Chen, Qiuxia Xiong

**Affiliations:** 1 Department of Oncology, Dazhou Central Hospital, Dazhou, 635000 Sichuan China; 2 School of Public Health, Kunming Medical University, Kunming, 650000 China; 3 Department of Radiotherapy, The Sixth Affiliated Hospital of Sun Yat-Sen University, Guangzhou, 510655 China; 4 Department of Nursing, Dazhou Vocational and Technical College, Dazhou, 635000 Sichuan China; 5 Department of Clinical Laboratory, The First Affiliated Hospital of Kunming Medical University, Kunming, 650118 China; 6 Yunnan Key Laboratory of Laboratory Medicine, Kunming, 650032 China; 7 Yunnan Province Clinical Research Center for Laboratory Medicine, Kunming, 650032 China

**Keywords:** Postoperative CEA, Recurrence risk, Stage II colon cancer, Adjuvant chemotherapy, Cancer, Oncology

## Abstract

Most clinical doctors rely on high-risk factors recommended by guidelines to decide whether to undergo adjuvant chemotherapy for stage II colon cancer. However, these high-risk factors do not include postoperative carcinoembryonic antigen (CEA). This study aims to explore the elevation of postoperative CEA as a risk factor, in addition to other high-risk factors, to guide adjuvant chemotherapy for patients with stage II colon cancer. A retrospective analysis was conducted on stage II colon cancer patients who underwent curative surgery at Yunnan Cancer Hospital and The Sixth Affiliated Hospital of Sun Yat-Sen University from April 2008 to January 2019. Patients were classified into three groups based on high-risk factors recommended by guidelines and postoperative CEA levels: low-risk with normal postoperative CEA, low-risk with elevated postoperative CEA and high-risk. COX regression analysis was used to identify independent prognostic factors affecting patients’ recurrence free survival (RFS). The Kaplan–Meier method was used to create the patients’ RFS curve. The restricted cubic spline (RCS) curve was used to assess the correlation between postoperative CEA and RFS on a continuous scale. Among 761 patients, there were 444 males (62.01%), with a median [IQR] age of 58.0 (18.0–88.0) years. A group of 425 high-risk patients had a 3-year RFS of 82.2% (95% CI 78.5–86.1%), while a group of 291 low-risk patients had a 3-year RFS of 89.7% (95% CI 86.1–93.5%). There was a statistically significant difference between the two groups (HR 1.83; 95% CI 1.22–2.74; P = 0.0067). Among them, the 3-year RFS of 261 low-risk patients with normal postoperative CEA was 93.6% (95% CI 90.5–96.8%), while the 3-year RFS of 30 low-risk patients with elevated postoperative CEA was 57.3% (95% CI 41.8–71.4%). There was a significant difference compared to the 3-year RFS of 425 high-risk patients (overall log-rank P < 0.0001). The multivariate analysis adjusted by the COX proportional hazards model showed that low-risk patients with elevated postoperative CEA patients (HR 14.95, 95% CI 4.51–49.63, P < 0.0001) was independently associated with a 3-year RFS. The restricted cubic spline model showed that in stage II colon cancer patients with tumor diameter > 1.955 ng/mL, the risk of postoperative recurrence increased with increasing postoperative CEA levels. Patients with elevated postoperative CEA levels have a significantly increased risk of recurrence. They should be included as high-risk factors to guide adjuvant chemotherapy for stage II colon cancer.

## Introduction

The incidence and mortality rates of colorectal cancer are increasing year by year worldwide^1^. It is estimated that as of January 1, 2022, there were over 1.4 million men and women in the United States with colorectal cancer, and 151,030 new cases of this disease will be diagnosed in 2022^2^. It poses a severe threat to the lives and health of people worldwide. Therefore, the diagnosis and treatment of colorectal cancer have become increasingly important. Guidelines^3–6^ recommend that the most important treatment for stage II colon cancer patients is curative surgical resection. However, some patients who undergo surgical resection are still at risk of recurrence and metastasis. Most clinicians mainly rely on high-risk factors recommended in the guidelines to decide whether to administer adjuvant chemotherapy to stage II colon cancer patients. Although CSCO^3^, ASCO^4^, ESMO^5^, and NCCN^6^ have identified high-risk factors for stage II colon cancer and recommended clinicians to consider adjuvant chemotherapy for patients with one or more of these high-risk factors, the currently recommended high-risk factors^3–6^ include pT4, less than 12 lymph node dissections, poor histological differentiation, bowel perforation or obstruction, lymphovascular invasion, neural invasion, positive circumferential resection margin, mucinous carcinoma and tumor budding.

Adjuvant chemotherapy for patients with stage II colon cancer is a controversial area in oncology. Adjuvant chemotherapy aims to eradicate micrometastatic disease present at the time of surgery, prevent the development of distant metastatic disease and thus cure those patients of their cancer. National and international guidelines for adjuvant therapy for stage II colon cancer recommend a range of treatment options from observation to single-agent or combination chemotherapy, depending on the presence of high-risk features. In a prospective study aimed at elucidating the role of adjuvant chemotherapy in stage II colon cancer^7^, it was observed that patients who received adjuvant chemotherapy had a slightly improved overall survival (OS) rate, which was statistically significant. However, although adjuvant chemotherapy may play a role in treating patients with stage II colon cancer, it is modest and associated with an increased risk of chemotherapy-related complications and death.

Although less than 10% of low-risk stage II colon cancer patients in the United States National Cancer Data receive adjuvant chemotherapy. Low-risk stage II colon cancer patients who receive adjuvant chemotherapy show improved survival outcomes at 1, 3, and 5 years, with a relative risk reduction in mortality of 12%^8^. Adjuvant chemotherapy is not routinely recommended for stage II colon cancer patients who do not belong to the high-risk subgroup. Therefore, it is essential to search for simple and effective prognostic indicators to predict the risk of postoperative recurrence in stage II colon cancer and guide whether adjuvant chemotherapy should be performed. However, these high-risk factors do not include carcinoembryonic antigen (CEA). Preoperative levels of CEA greater than 5 ng/mL or an increase in detected levels are associated with colon cancer recurrence^9^. It is the most widely used prognostic indicator for colon cancer to date^10^. In our latest study^11^, 2160 colorectal cancer patients from three hospitals in China were enrolled. Preoperative CEA is not as effective as other risk factors in predicting colon cancer prognosis and cannot be used as a sole prognostic indicator for postoperative recurrence of colon cancer. Because some patients with elevated preoperative CEA levels return to normal after curative surgery, their prognosis needs to be evaluated in conjunction with postoperative CEA levels. Therefore, postoperative CEA is more important than preoperative CEA^12^.

The aim of this study was to investigate the elevation of postoperative CEA as a risk factor for guiding adjuvant chemotherapy in stage II colon cancer patients, independent of other high-risk factors. Furthermore, we aimed to validate whether the prognostic impact of postoperative CEA depends on other high-risk factors.

## Methods

### Ethics approval and consent to participate

The Ethics Committees of Yunnan Cancer Hospital (No. KY201824) and the Sixth Affiliated Hospital of Sun Yat-sen University (No. 2021ZSLYEC-051) have approved this retrospective study. The study adheres to the Helsinki Declaration and the Guidelines for Good Clinical Practice. Due to its retrospective nature, the requirement for informed consent was waived by the ethics Committees of Yunnan Cancer Hospital and the Sixth Affiliated Hospital of Sun Yat-sen University. All patient data in the investigation were anonymous. Written informed consent was obtained from all patients.

### Study design and patient cohort

**Figure 1:**
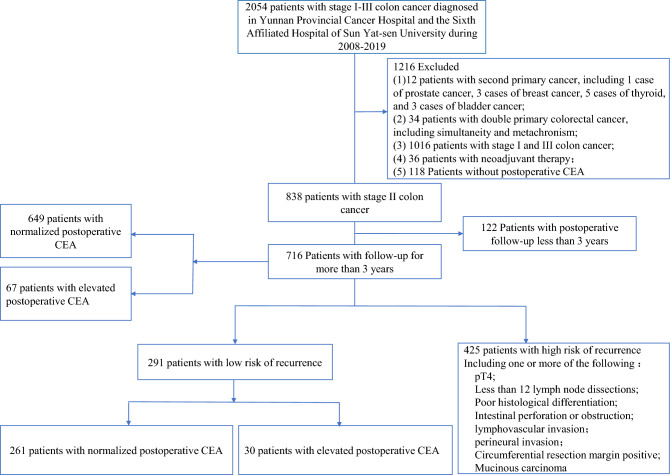
Study flow chart.

According to the STROBE guidelines^13^, we retrospectively included 761 stage II colon cancer patients who underwent curative surgery from April 2008 to February 2019 at either Yunnan Cancer Hospital or the Sixth Affiliated Hospital of Sun Yat-sen University. Please refer to Fig. 1 for the study flowchart and detailed inclusion and exclusion criteria. Extract the CEA value closest to the surgery time from the electronic medical record. Postoperative CEA is defined as the final CEA value within 12 weeks after surgery or before the start of adjuvant chemotherapy (12). All CEA measurements at Yunnan Cancer Hospital are performed using the COBAS 800 e602 immunoassay analyzer (Roche Diagnostics, Tokyo, Japan) and chemiluminescent immunoassay analyzer. The Sixth Affiliated Hospital of Sun Yat-sen University uses the Alinity I immunoassay analyzer (Abbott Diagnostics, Chicago, USA) following the WHO standard method (code 73/601). The reference range for serum CEA is 0.0 to 5.0 ng/mL. Values above 5.0 ng/mL are considered elevated CEA, while values below 5.0 ng/mL are considered normal.

Meanwhile, collect demographic, clinical, and pathological data from patients. Extracted variables include age, gender, body mass index (BMI), pre- and postoperative serum carcinoembryonic antigen (CEA) levels, serum carbohydrate antigen 19-9 (CA19-9) levels, neutrophil-to-lymphocyte ratio (NLR), primary site (right or left colon), surgical approach (open or laparoscopic resection), tumor differentiation grade (well, moderately, or poorly differentiated), pathological T stage (T3 or T4), Lymph node yield (≥ 12 or < 12), mucinous type (yes or no), circumferential resection margin(positive or negative), adjuvant chemotherapy(yes or no), chemotherapy regime (fluorouracil [FU]/capecitabine, CAPOX/XELOX, FOLFOX, or other), chemotherapy cycles (< 6 or ≥ 6 cycles).

These risk factors include pT4, < 12 lymph node dissections, poor histological differentiation, bowel perforation or obstruction, lymphovascular invasion, neural invasion, positive CRM, mucinous carcinoma. The high-risk group is defined as patients who have one or multiple risk factors simultaneously. The low-risk group is defined as other patients who do not have any high-risk factors.

### Exposures

Divide patients into three groups: the low-risk with normal postoperative CEA group, the low-risk with elevated postoperative CEA group, and the high-risk group.

### Surveillance protocol

The clinical evaluation of the patient includes serum CEA level detection, physical examination, imaging examinations (CT/MRI/PET-CT), and colonoscopic biopsy. CEA levels should be measured every 3 to 6 months for a continuous period of 3 years. Imaging examinations, including plain and contrast-enhanced scans of the patient’s chest, abdomen, and pelvis, should be performed at least once every 12 months or at least once every 3 years. Colonoscopy is performed once a year after surgery, and once every 3 years thereafter. Colonoscopy, histological examination or imaging examination confirms whether there is recurrence or distant metastasis in all cases.

### Outcomes

This study combines postoperative CEA levels to predict and evaluate the likelihood and value of recurrence in colon cancer patients after radical surgery. It is worth noting that disease-free survival refers to the time from surgery until the patient experiences recurrence, metastasis, or death. If a patient is lost to follow-up, the recurrence free survival (RFS) will be calculated based on the date of the last follow-up. All enrolled patients received a complete 3-year follow-up, and those who did not complete 3 years were not included in this study.

### Statistical analysis

Continuous variables were presented as mean ± standard deviation (SD) for normally distributed data or median (interquartile range) for skewed data. Categorical variables were presented as frequency or percentage. Chi-square or Fisher’s exact test (for discrete variables) and unpaired t-test, Wilcoxon signed-rank test, or analysis of variance (ANOVA) for continuous variables were used to compare patient characteristics. Survival analysis was conducted using the Kaplan–Meier method and log-rank test. All P values below 0.05 were statistically significant. The COX proportional hazards regression model was used to evaluate factors independently associated with RFS. Variables included in the final multivariable model were selected based on their clinical relevance and statistical significance in univariate analysis (cutoff value, P < 0.05). Intestinal obstruction or perforation, and positive CRM were not included in the multivariable analysis due to their low positivity rates. The correlation between postoperative CEA and RFS was evaluated on a continuous scale using restricted cubic splines (RCS) curves. Subgroup analysis was performed based on known risk factors, and interaction tests were conducted through the COX regression model. The internal validation of the final multivariate model for RFS was performed through a bootstrap sampling procedure (n = 1000 samples) on a population with an overall recurrence risk score. Statistical analysis was conducted using R software (version 3.6.3; http://www.R-project.org), SPSS 28.0, and GraphPad Prism 8 for plotting^13^.

## Results

A total of 2054 patients with stage I to III rectal cancer who underwent surgical resection at Yunnan Cancer Hospital and Sixth Affiliated Hospital of Sun Yat-sen University from 2008 to 2019 were retrospectively collected, and 1338 patients were excluded (see Fig. 1 for inclusion and exclusion criteria). Finally, 716 patients with stage II colorectal cancer were included. Patients were divided into 2 groups according to guideline-recommended risk factors (3–6), of which 291 were low-risk patients and 425 were high-risk patients. Of the 291 low-risk patients, 261 had normal postoperative CEA and 30 had elevated postoperative CEA (Fig. 1). Of the 761 patients, 444 (62.01%) were male, and the median [IQR] age was 58.0 (18.0–88.0) years. The follow-up time exceeded 3 years, and they met the inclusion criteria. There were 97 cases of local recurrence and distant metastases, with a recurrence rate of 12.75%. The median follow-up time was 49.73 (95% CI 45.73–51.10) months. The clinicopathological characteristics are shown in Table 1.

**Table 1:** Baseline characteristics.

Variable	Total (n = 716)	Postoperative CEA group		P-value
		Normal postoperative CEA (n = 649)	Elevated postoperative CEA (n = 67)	
Preoperative CEA, ng/mL				
Mean (SD)	11.95 (29.04)	7.62 (13.13)	53.39 (73.79)	< 0.001
Median (IQR)	3.53 (0.20–356.50)	3.14 (0.20–4.89)	23.51 (5.66–356.50)	
Preoperative CA199, ng/mL				
Mean (SD)	34.54 (97.99)	32.96 (92.10)	49.54 (142.34)	0.188
Median (IQR)	12.23 (0.60–1145.51)	12.04 (0.60–1145.51)	17.67 (0.60–1101.94)	
Preoperative NLR				
Mean (SD)	3.02 (2.85)	2.99 (2.88)	3.35 (2.56)	0.410
Median (IQR)	2.29 (0.06–26.89)	2.27 (0.06–26.89)	2.60 (1.06–14.13)	
Postoperative CEA				
Mean (SD)	3.72 (12.43)	2.00 (1.04)	20.37 (36.78)	< 0.001
Median (IQR)	1.96 (0.30–205.60)	1.79 (0.30–5.87)	7.64 (5.11–205.60)	
Postoperative CA199				
Mean (SD)	16.59 (74.36)	12.03 (14.08)	60.77 (236.17)	< 0.001
Median (IQR)	8.96 (0.60–1472.00)	8.82 (0.60–210.90)	11.95 (0.60–1472.00)	
Postoperative NLR				
Mean (SD)	2.02 (1.76)	1.98 (1.76)	2.34 (1.84)	0.192
Median (IQR)	1.65 (0.01–23.80)	1.64 (0.01–23.80)	1.80 (0.61–10.64)	
Age (years)				
Mean (SD)	57.13 (12.01)	56.65 (11.95)	61.79 (11.73)	< 0.001
Median (IQR)	58.00 (18.00–88.00)	58.00 (18.00–88.00)	63.00 (34.00–85.00)	
BMI (kg/m^2^)				
Mean (SD)	22.56 (3.31)	22.63 (3.31)	21.85 (3.34)	0.111
Median (IQR)	22.31 (13.89–40.40)	22.32 (13.89–40.40)	21.26 (15.62–28.91)	
Hospital, n (%)				0.607
YNCH	542 (75.70%)	493 (75.96%)	49 (73.13%)	
SYSU6	174 (24.30%)	156 (24.04%)	18 (26.87%)	
Sex, no. (%) of patients				0.239
Male	444 (62.01%)	398 (61.33%)	46 (68.66%)	
Female	272 (37.99%)	251 (38.67%)	21 (31.34%)	
Surgical approach				0.103
OR	436 (60.89%)	389 (59.94%)	47 (70.15%)	
LR	280 (39.11%)	260 (40.06%)	20 (29.85%)	
Tumor differentiation				0.556
Unknown	31 (4.33%)	27 (4.16%)	4 (5.97%)	
Well	36 (5.03%)	33 (5.08%)	3 (4.48%)	
Moderate	463 (64.66%)	416 (64.10%)	47 (70.15%)	
Poor-undifferentiated	186 (25.98%)	173 (26.66%)	13 (19.40%)	
Mucinous type				0.905
No	512 (72.93%)	465 (73.00%)	47 (72.31%)	
Yes	190 (27.07%)	172 (27.00%)	18 (27.69%)	
T stage				0.254
T3	632 (88.27%)	570 (87.83%)	62 (92.54%)	
T4	84 (11.73%)	79 (12.17%)	5 (7.46%)	
Lymph node yield				0.418
< 12	650 (90.78%)	591 (91.06%)	59 (88.06%)	
≥ 12	66 (9.22%)	58 (8.94%)	8 (11.94%)	
LVI				0.506
No	683 (95.39%)	618 (95.22%)	65 (97.01%)	
Yes	33 (4.61%)	31 (4.78%)	2 (2.99%)	
PNI				0.116
No	673 (94.26%)	607 (93.82%)	66 (98.51%)	
Yes	41 (5.74%)	40 (6.18%)	1 (1.49%)	
Adjuvant chemotherapy				0.202
No	130 (18.16%)	114 (17.57%)	16 (23.88%)	
Yes	586 (81.84%)	535 (82.43%)	51 (76.12%)	
Primary site				0.840
Right colon	387 (54.05%)	350 (53.93%)	37 (55.22%)	
Left colon	329 (45.95%)	299 (46.07%)	30 (44.78%)	
Intestinal perforation/obstruction				0.537
No	710 (99.16%)	644 (99.23%)	66 (98.51%)	
Yes	6 (0.84%)	5 (0.77%)	1 (1.49%)	
CRM				0.577
No	713 (99.58%)	646 (99.54%)	67 (100.00%)	
Yes	3 (0.42%)	3 (0.46%)	0 (0.00%)	
Chemotherapy regimen				0.452
5-FU/capecitabine	107 (18.26%)	97 (18.13%)	10 (19.61%)	
CAPOX/XELOX	163 (27.82%)	145 (27.10%)	18 (35.29%)	
FOLFOX	290 (49.49%)	270 (50.47%)	20 (39.22%)	
Other	26 (4.44%)	23 (4.30%)	3 (5.88%)	
Chemotherapy cycle				0.144
< 6	276 (47.10%)	247 (46.17%)	29 (56.86%)	
≥ 6	310 (52.90%)	288 (53.83%)	22 (43.14%)	

Data are presented as median (IQR), mean (SD), or n (%).

*BMI* body mass index, *CEA* carcinoembryonic antigen, *CA 19-9* carcinoma antigen 19-9, *NLR* neutrophil lymphocyte ratio, *LR* laparoscopic resection, *LVI* lymphovascular invasion, *OR* open resection, *PNI* perineural invasion, *YNCH* Yunnan Cancer Hospital, *SYSU6* the Sixth Affiliated Hospital of Sun Yat-sen University, *CRM* circumferential resection margin.

P value, using Wilcoxon Mann–Whitney test, Chi-square test, or Fisher’s exact test, depending on whether the variable is continuous or categorical.

### Kaplan–Meier analysis of different groups

**Figure 2:**
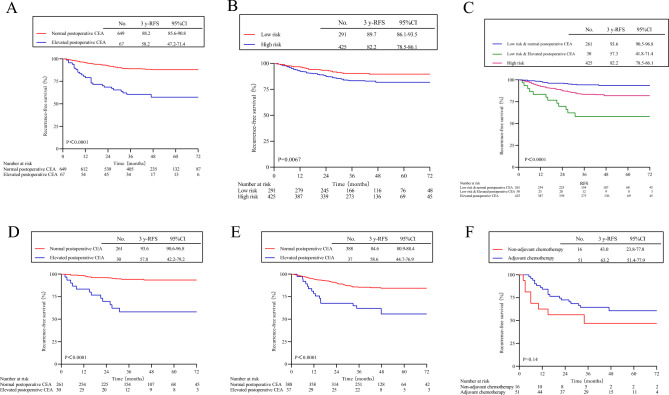
Kaplan–Meier curves of recurrence-free survival based on different grouping methods. (**A**) Postoperative normal CEA vs postoperative elevated CEA in the overall patient population. (**B**) High-risk vs low-risk in the overall patient population. (**C**) low-risk patients with postoperative normal CEA vs low-risk patients with postoperative elevated CEA vs high-risk in the overall patient population. (**D**) Postoperative normal CEA vs postoperative elevated CEA in low-risk patients. (**E**) Postoperative normal CEA vs postoperative elevated CEA in high-risk patients. (**F**) Adjuvant chemotherapy with postoperative elevated CEA vs Non-adjuvant chemotherapy.

**Figure 3:**
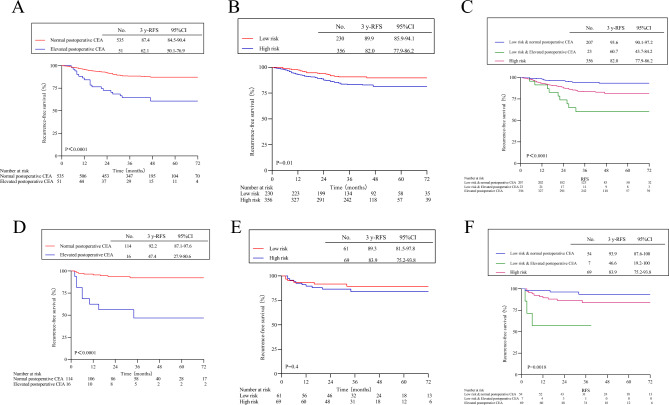
Kaplan–Meier survival curves of recurrence-free survival rates based on different grouping methods in chemotherapy and non-chemotherapy groups. (**A**) Postoperative normal CEA vs postoperative elevated CEA in the adjuvant chemotherapy population. (**B**) High-risk vs low-risk in the adjuvant chemotherapy population. (**C**) low-risk patients with postoperative normal CEA vs low-risk patients with postoperative elevated CEA vs high-risk in the adjuvant chemotherapy population. (**D**) Postoperative normal CEA vs postoperative elevated CEA in the non-adjuvant chemotherapy. (**E**) High-risk vs low-risk in the non-adjuvant chemotherapy population. (**F**) low-risk patients with postoperative normal CEA vs low-risk patients with postoperative elevated CEA vs high-risk in the non-adjuvant chemotherapy population.

The 3-year RFS of 649 postoperative patients with normal CEA was 88.2% (95% CI 85.6–90.8%), while the 3-year RFS of 67 postoperative patients with elevated CEA was 58.2% (95% CI 47.2–71.4%). There was a statistically significant difference between the two groups (HR 4.28; 95% CI 2.08–8.81; P < 0.0001) (Fig. 2A). In the low-risk population (Fig. 2D), high-risk population (Fig. 2E), chemotherapy group (Fig. 3A), and non-chemotherapy group (Fig. 3D), there were statistically significant differences between the two groups of patients, leading to similar results.

The 3-year RFS of 425 high-risk patients was 82.2% (95% CI 78.5–86.1%), while that of 291 low-risk patients was 89.7% (95% CI 86.1–93.5%). There was a statistically significant difference between the two groups (HR 1.83; 95% CI 1.22–2.74; P = 0.0067) (Fig. 2B). In the chemotherapy population (Fig. 3B), there was a statistically significant difference among the two patient groups, but no significant difference was observed among patients without chemotherapy (HR 1.53; 95% CI 0.58–4.09; P = 0.4) (Fig. 3E).

The 3-year RFS was 93.6% (95% CI 90.5–96.8%) for 261 low-risk patients with normal postoperative CEA, and 57.3% (95% CI 41.8–71.4%) for 30 low-risk patients with elevated postoperative CEA, showing a statistically significant difference compared to the 3-year RFS of 425 high-risk patients (overall log-rank P < 0.0001) (Fig. 2C). In both the chemotherapy group (Fig. 3C) and the non-chemotherapy group (Fig. 3F), there was a statistically significant difference among the three groups of patients, leading to similar results.

Among the 67 patients with postoperative CEA elevation, those who received adjuvant chemotherapy (n = 51) had a 20.2% reduction in recurrence risk compared to those without chemotherapy (n = 16) (43.0%, 95% CI 23.8–77.8% vs. 63.2%, 95% CI 51.4–77.9%). However, it did not reach statistical significance (HR 0.55, 95% CI 0.21–1.44, P = 0.14) (Fig. 2F).

### Multivariate analyses of all variables

**Figure 4:**
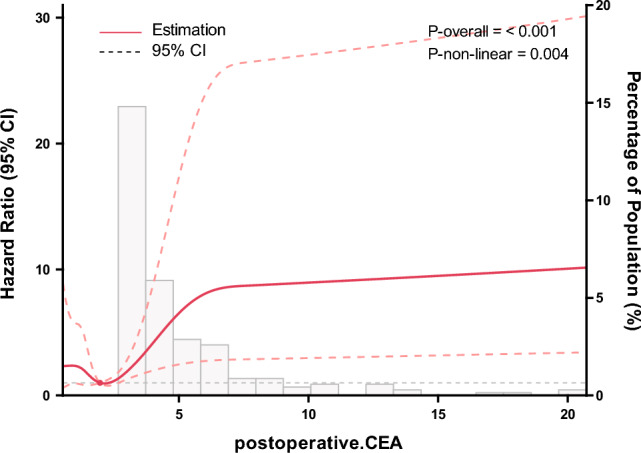
The relationship between postoperative CEA as a continuous variable and hazard ratio for recurrence. The red solid line represents the unadjusted hazard ratio, and the red dashed line represents the 95% confidence interval obtained from restricted cubic spline regression.

**Figure 5:**
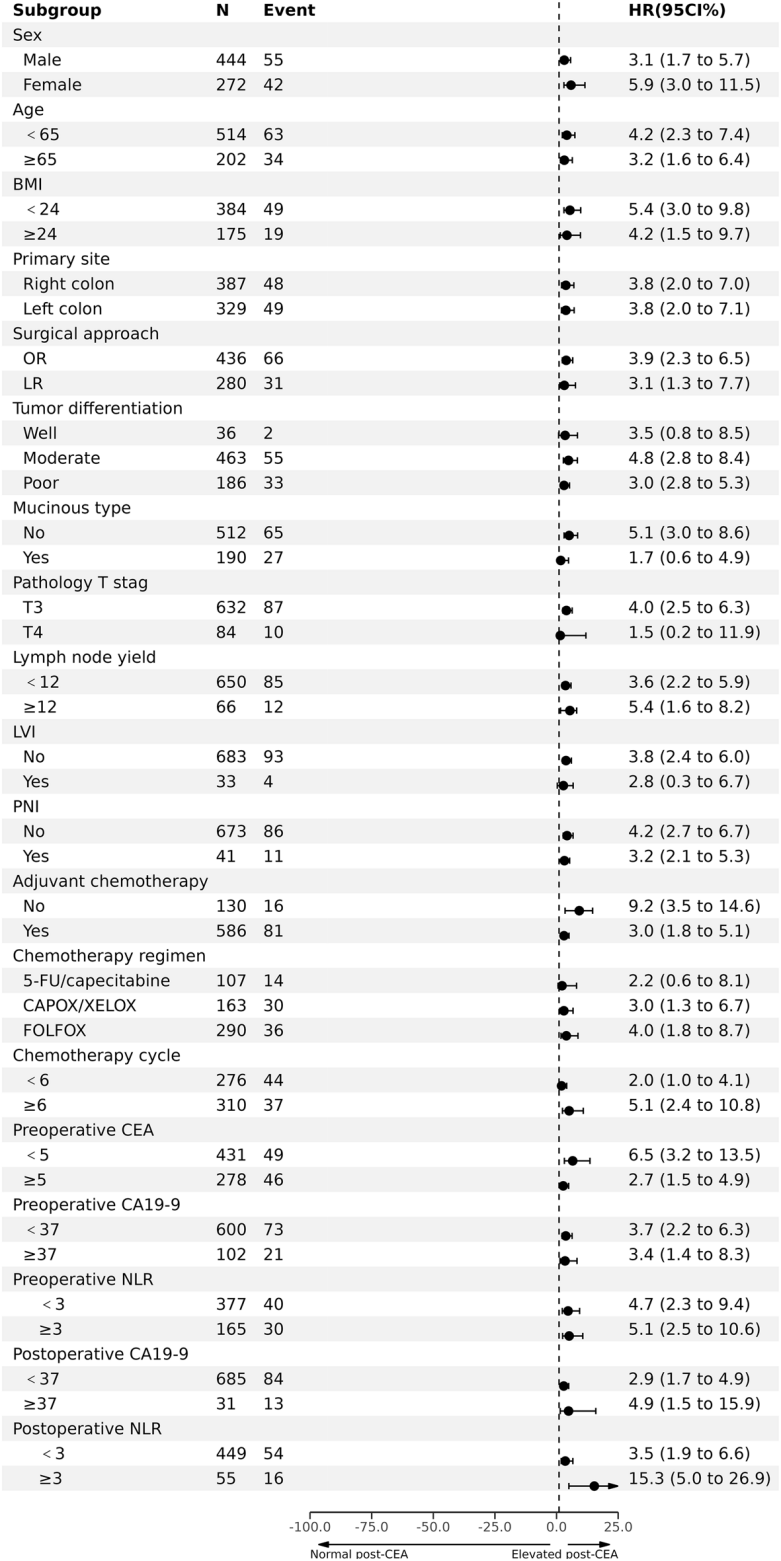
Forest plot of recurrence-free survival in the preoperative CEA group, stratified by clinicopathological characteristics based on Cox model. P-values for interaction were calculated using Cox regression model. HR and 95% CI were presented with squares and error bars. *CI* confidence interval, *HR* hazard ratio, *RFS* recurrence-free survival.

Table 2 shows the univariate and multivariate analysis of factors related to RFS. In the univariate analysis, neural invasion, preoperative elevation of CEA and CA199, preoperative NLR ≥ 3, postoperative elevation of CEA and CA199, and postoperative NLR ≥ 3 were associated with shortened RFS (P < 0.05). Multivariate analysis showed that postoperative elevations of CEA (HR 4.79, 95% CI 2.65–8.65, P < 0.0001) and CA199 (HR 2.69, 95% CI 1.18–6.13, P = 0.0189) were independently associated with shorter RFS. After adjusting for confounding factors and incorporating multiple COX models, postoperative elevation of CEA in low-risk patients (HR, 14.95; 95% CI 4.51–49.63; P < 0.0001) was independently associated with 3-year RFS (Table 3). A restricted cubic spline model showed that the risk of recurrence after surgery increased with increasing postoperative CEA levels in stage II colon cancer patients with tumor diameter > 1.955 ng/mL (Fig. 4). Subgroup analysis of RFS also found that postoperative elevation of CEA was independently associated with RFS, without interaction with other known clinicopathological factors related to prognosis (Fig. 5).

**Table 2:** Univariate and multivariate analyses of 3-year recurrence free survival.

Variables	Univariate		Multivariate	
	HR (95% CI)	P-value	HR (95% CI)	P-value
Sex				
Male	1.0 (reference)			
Female	1.29 (0.87, 1.93)	0.2104		
Age group				
< 65	1.0 (reference)			
≥ 65	1.40 (0.92, 2.13)	0.1135		
BM group				
< 24	1.0 (reference)			
≥ 24	0.83 (0.49, 1.42)	0.5001		
Primary site				
Right colon	1.0 (reference)			
Left colon	1.20 (0.81, 1.79)	0.3690		
Surgical approach				
OR	1.0 (reference)			
LR	0.73 (0.48, 1.12)	0.1530		
Tumor differentiation				
Well	1.0 (reference)			
Moderate	0.21 (0.04, 1.00)	0.0503		
Poor-undifferentiated	0.48 (0.22, 1.06)	0.0710		
Mucinous type				
No	1.0 (reference)			
Yes	1.10 (0.70, 1.72)	0.6806		
Pathology T stage				
T3	1.0 (reference)			
T4	0.83 (0.43, 1.60)	0.5794		
Lymph node yield				
< 12	1.0 (reference)			
≥ 12	1.37 (0.75, 2.52)	0.3074		
LVI				
No	1.0 (reference)			
Yes	0.84 (0.31, 2.30)	0.7399		
PNI				
No	1.0 (reference)		1.0 (reference)	
Yes	2.16 (1.15, 4.05)	0.0164	2.55 (0.35, 18.73)	0.3565
Adjuvant chemotherapy				
No	1.0 (reference)			
Yes	1.02 (0.60, 1.75)	0.9298		
Chemotherapy regimen				
5-FU/capecitabine	1.0 (reference)			
CAPOX/XELOX	1.40 (0.74, 2.64)	0.2995		
FOLFOX	0.92 (0.49, 1.70)	0.7854		
Other	0.26 (0.03, 2.00)	0.1965		
Chemotherapy cycle				
< 6	1.0 (reference)			
≥ 6	0.75 (0.48, 1.16)	0.1977		
Preoperative CEA				
< 5	1.0 (reference)		1.0 (reference)	
≥ 5	1.54 (1.03, 2.30)	0.0365	0.75 (0.43, 1.30)	0.3057
Preoperative CA19-9				
< 37	1.0 (reference)		1.0 (reference)	
≥ 37	1.82 (1.12, 2.97)	0.0154	1.04 (0.51, 2.11)	0.9103
Preoperative NLR				
< 3	1.0 (reference)		1.0 (reference)	
≥ 3	1.83 (1.14, 2.94)	0.0125	1.55 (0.94, 2.56)	0.0857
Postoperative CEA				
< 5	1.0 (reference)		1.0 (reference)	
≥ 5	3.79 (2.43, 5.90)	< 0.0001	4.79 (2.65, 8.65)	< 0.0001
Postoperative CA19-9 group				
< 37	1.0 (reference)		1.0 (reference)	
≥ 37	4.46 (2.48, 8.01)	< 0.0001	2.69 (1.18, 6.13)	0.0189
Postoperative NLR				
< 3	1.0 (reference)		1.0 (reference)	
≥ 3	2.47 (1.41, 4.32)	0.0015	1.62 (0.86, 3.05)	0.1394

*BMI* body mass index, *CEA* carcinoembryonic antigen, *CA 19–9* carcinoma antigen 19–9, *NLR* neutrophil lymphocyte ratio, *LR* laparoscopic resection, *LVI* lymphovascular invasion, *OR* open resection, *PNI* perineural invasion.

**Table 3:** Adjusted hazard ratios of 3-year RFS according to the new risk factor group.

New risk factor group	N	Events (%)	Model 1		Model 2		Model 3	
			HR (95% CI)	*P*-value	HR (95% CI)	P value	HR (95% CI)	*P*-value
Low risk and normal postoperative CEA	261	17 (36.50)	1.0 (Ref.)		1.0 (Ref.)		1.0 (Ref.)	
Low risk and elevated postoperative CEA	30	12 (3.51)	7.35 (3.51, 15.40)	< 0.0001	16.89 (6.62, 43.15)	< 0.0001	14.95 (4.51, 49.63)	< 0.0001
High risk	425	68 (56.99)	2.56 (1.51, 4.36)	0.0005	4.16 (1.97, 8.78)	0.0002	2.95 (1.17, 7.47)	0.0225

*HR* hazard ratios, *Ref.* reference.

^a^Model 1 was unadjusted.

^b^Model 2 was adjusted for age (< 65 vs. ≥ 65), body mass index (< 24 vs. ≥ 24), and sex (male vs. female).

^c^Model 3 was adjusted for age (< 65 vs. ≥ 65), body mass index (< 24 vs. ≥ 24), sex (male vs. female), surgical approach (open resection vs. laparoscopic resection), location (right colon vs. left colon), adjuvant chemotherapy (yes vs. no), chemotherapy regimen (5-FU/capecitabine vs. CAPOX/XELOX vs. FOLFOX vs. other), chemotherapy cycle (< 6 vs. ≥ 6), preoperative CEA, ng/mL (≤ 5 vs. > 5), preoperative CA19-9, ng/mL (≤ 37 vs. > 37), postoperative NLR (< 3 vs. ≥ 3), postoperative CA19-9, ng/mL (≤ 37 vs. > 37), and postoperative NLR(< 3 vs. ≥ 3).

## Discussion

There are limitations to identifying risk factors for guiding adjuvant chemotherapy in stage II colon cancer patients. This study suggests incorporating postoperative CEA levels as a risk factor to assess the risk of recurrence and guide chemotherapy. In multivariate analysis, postoperative elevation of CEA was identified as an independent prognostic parameter that may affect treatment decisions even in the absence of other risk factors. This study incorporated potential risk factors for colon cancer recurrence into a COX proportional hazards model and identified two independent risk factors: postoperative CEA and postoperative CA199. It is worth noting that preoperative elevation of CEA is not an independent risk factor for 3-year disease-free survival in stage II colon cancer patients, which is consistent with previous studies^12^. Using only the TNM staging system for prognostic stratification of colon cancer has some limitations. The internationally recognized serum CEA is an important prognostic indicator for colorectal cancer^14^. Postoperative CEA is an independent risk factor for 3-year recurrence-free survival in stage II colon cancer patients. A study^15^ found that postoperative positive CEA and CEA increment were independent prognostic factors for stage II colon cancer. Patients with elevated postoperative CEA levels and positive CEA increments had the worst PFS and OS compared to other groups. The results of this study can provide reference for adjuvant therapy in stage II rectal cancer after radical surgery. Prognostic factors are not only related to pathological staging (T4 and/or N2), but also to preoperative high CEA levels. The combination of pT, pN, and preoperative high CEA levels may be predictive factors for resistance to CapeOX adjuvant chemotherapy^16^. According to a study^17^, T4 infiltration, vascular infiltration, postoperative CEA level, and the number of lymph nodes removed during surgery may significantly affect the prognosis of patients with stage II CRC after radical resection. The risk of early postoperative recurrence and clinical outcome deterioration increases proportionally with the values of these four parameters. Studies^18,19^ have attempted to improve the accuracy of stratifying stage III colon cancer patients by constructing a prognostic model that combines postoperative CEA with TNM. However, this is only applicable to stage III colon cancer patients. It is currently unclear whether it is applicable to stage II colon cancer patients.

Numerous previous studies have reported risk factors for postoperative recurrence in stage II colon cancer patients, but no positive results were found in this study except for elevated postoperative levels of CEA and CA199. Preoperative NLR was correlated with RFS and OS, indicating that NLR can be used as a tool to determine which patients should receive/avoid adjuvant chemotherapy, especially for left-sided colon cancer. Based on receiver operating characteristic (ROC) curve analysis, the cutoff value of NLR was 3^20^. There are studies reporting that an NLR cutoff value of 5 is used for prognosis analysis^21^. The left colon is also a risk factor for the recurrence of stage II colon cancer after surgery^22^, especially in patients with MSS^23^. Special attention should be paid during follow-up. Mucinous histology may be an indicator for improving survival in stage II colon cancer chemotherapy^24^. There is also evidence that there is no significant difference in tumor-specific survival between adenocarcinoma and signet ring cell carcinoma. Stage II signet ring cell carcinoma should not receive adjuvant chemotherapy^25^. The overall survival (OS) of stage II colon cancer with less than 8 cleared lymph nodes is poor^26^. Studies recommend clearing 20 or more lymph nodes for accurate postoperative staging^27^. Adjuvant chemotherapy should be considered during the treatment of stage III colon cancer patients aged 70 or above, but chemotherapy has limited efficacy for stage II colon cancer in elderly patients^28^. Given the increasing incidence of colon cancer in young patients, doctors are more aggressive in treating stage II colon cancer. However, evidence for this treatment is limited^29^, and over-treatment leading to treatment-related harm should be avoided. The OS of patients with stage II colon cancer who underwent laparoscopic radical surgery is superior to those who underwent open radical surgery, especially for patients aged 75 or older^30^. In the largest group of stage II colon cancer patients evaluated so far^31^, regardless of treatment regimen, patient age, or high-risk pathological features, OS improvement is associated with adjuvant chemotherapy. The toxicity of the 3-month group was significantly lower than that of the 6-month group in chemotherapy cycle studies. Both 3-month CAPOX and 6-month FOLFOX can be used to treat stage II colorectal cancer patients^32^. The TOSCA trial confirmed that there was no significant difference in OS between the two groups. Compared with 5-FU/LV, FOLFOX is unlikely to be cost-effective^33^. Recent research^34^ has shown that a 3-month CAPOX regimen can be an effective treatment option. The convenience, reduced toxicity, and cost of using CAPOX as an adjuvant for 3 months suggest it as a potential option for high-risk stage II colon cancer^35^. However, adjuvant chemotherapy did not significantly improve cancer-specific survival in patients with adverse features of stage II colon cancer. Other markers are needed to select appropriate patients for adjuvant therapy^36^.

Two important risk factors, mismatch repair (MMR) gene expression and tumor budding (TB), were not included in this study. Previous studies did not find dMMR to have prognostic value in terms of overall and disease-free survival in patients with stage II colon cancer. The recurrence rate in patients with dMMR tumors was significantly reduced^37^. The survival rate of stage II dMMR colon cancer patients with high-risk factors is similar to that of patients without high-risk factors, regardless of the presence of KRAS mutations^38^. This study suggests that tumors with a pathological indicator of TB ≥ 5 may exhibit a high risk of recurrence and poor prognosis. The evaluation of TB may help identify patients suitable for neoadjuvant therapy^39^. The TB grading based on the ITBCC2016 criteria should be routinely evaluated in pathological practice and may improve the benefit of adjuvant chemotherapy for stage II colon cancer^40^.

There are limitations to this exploratory study. Firstly, due to its retrospective design, there were differences in the timing of postoperative CEA measurements. However, we selected values that were closest to the time of surgery. Patients who received adjuvant treatment beyond 12 weeks or received adjuvant treatment during the trial were excluded. Secondly, the limitations of this retrospective study include the lack of incorporation of mismatch repair gene status and tumor budding, which are important indicators. However, in the ESMO^5^ and CSCO^3^ guidelines, the population with high microsatellite instability caused by mismatch repair gene deficiency is small, and we prioritize T4 stage over high microsatellite instability. This study will continue to include more cases and wait for subsequent results to be published.

## Conclusions

Patients with elevated postoperative CEA levels have a significantly increased risk of recurrence. Although the proportion of patients with postoperative CEA elevation and no high-risk factors is low, they should still be considered as high-risk factors to guide adjuvant chemotherapy after surgery for stage II colon cancer.

## Data Availability

Original data are available upon request to the corresponding author, Q. Xiong.

## Abbreviations

RFS: Recurrence-free survival
CI: Confidence interval
HR: Hazard ratio
IQR: Interquartile range
CEA: Carcinoembryonic antigen
CA 19-9: Carcinoma antigen 19-9
NLR: Neutrophil lymphocyte ratio
LVI: Lymphovascular invasion
PNI: Perineural invasion
